# Turbulence-Resistant FSO Communication Using a Few-Mode Pre-Amplified Receiver

**DOI:** 10.1038/s41598-019-52698-1

**Published:** 2019-11-07

**Authors:** Huiyuan Liu, Bin Huang, Juan Carlos Alvarado Zacarias, He Wen, Haoshuo Chen, Nicolas K. Fontaine, Roland Ryf, Jose Enrique Antonio-Lopez, Rodrigo Amezcua Correa, Guifang Li

**Affiliations:** 1 CREOL, The College of Optics & Photonics, University of Central Florida, Orlando, FL 32816 USA; 2 Nokia Bell Labs, 791 Holmdel Rd, Holmdel, NJ 07733 USA

**Keywords:** Adaptive optics, Fibre optics and optical communications

## Abstract

Leveraging recent advances in space-division multiplexing, we propose and demonstrate turbulence-resistant free-space optical communication using few-mode (FM) pre-amplified receivers. The rationale for this approach is that a distorted wavefront can be decomposed into a superposition of the fundamental Gaussian mode and high-order modes of a few-mode fiber. We present the noise statistics and the sensitivity of the FM pre-amplified receiver, followed by experimental and numerical comparisons between FM pre-amplified receivers and single-mode (SM) pre-amplified receivers with or without adaptive optics. FM pre-amplified receivers for FSO can achieve high sensitivity, simplicity and reliability.

## Introduction

**Figure 1:**
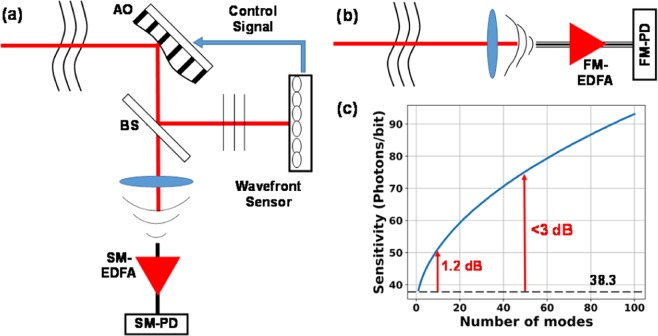
Schematic of FSO using (**a**) adaptive optics, (**b**) a FM pre-amplified receiver with (**c**) its sensitivity as a function of the number of modes.

Free-space optical (FSO) communication offers an orders-of-magnitude increase in transmission capacity compared to that of the radio-frequency technology, through the air or water^1,2^. Unfortunately, atmospheric turbulence distorts the wavefront, resulting in spatiotemporal amplitude and phase fluctuations at the detector^3^. Current FSO communication is dominated by the use of adaptive optics (AO) to correct wavefront distortions^4,5^, followed by single-mode (SM) optically pre-amplified receivers, as shown in Fig. 1(a). If wavefront correction is perfect, such a system can restore the ideal receiver sensitivity at 38.3 photons/bit for on-off keying (OOK) modulation^6^. However, AO FSO systems are expensive and have large size, weight, as well as power consumption. Yet, AO FSO systems still leave much to be desired in terms of performance and reliability. The splitting loss for wavefront sensing dictates that the above theoretical sensitivity limit cannot be achieved in practice. Furthermore, a single AO cannot correct both phase and amplitude distortions associated with moderate and strong turbulence. Since reliability is the key to widespread adoption of FSO communication, it is highly desirable to develop alternative approaches to combat turbulence and improve FSO reliability.

Figure 1(b) illustrates the schematic of our proposal, in which the complicated AO and the SM photodetector are replaced by a few-mode (FM) amplifier, which became available very recently due to advances in space-division multiplexing (SDM), and the FM photodetector, respectively^7,8^. The incoming distorted wavefront can be decomposed into the fundamental Gaussian mode and high-order modes; the stronger the turbulence the more spatial modes. Fibers with larger numerical apertures (NAs) and/or larger cores can reduce coupling loss, so the signal contained in the distorted wavefront can be received in its entirety by a FM photodiode without pre-amplification. However, such a receiver would lose advantages of optical pre-amplification. Specifically, the sensitivity of receivers based solely on a FM photodiode will be thermal noise limited while that of an optical pre-amplified receiver will instead be limited by noises associated with amplified spontaneous emission (ASE). For SM OOK receivers, the thermal noise-limited sensitivity is >6000 photons/bit while the signal-ASE beat noise-limited sensitivity is 38.3 photons/bit at 10 Gb/s^6^. Therefore, the FM pre-amplifier in Fig. 1(b) is essential for constructing a simplified receiver while maintaining high sensitivity.

## Noise Statistics And Sensitivity Of FM Pre-Amplified PD

We first obtain the sensitivity of FM pre-amplified receivers based on the noise statistics of the photocurrent. For a FM pre-amplified photodetector, the total optical signal can be written as

1 $$S(x,y,t)=\mathop{\sum }\limits_{m=1}^{M}{E}_{{v}_{0},m}{{\rm{\psi }}}_{m}(x,y){e}^{j2\pi {v}_{0}t+j{\theta }_{m}}$$

where *M* is the number of modes received by the detector, *E*_*v*0_,*m* is the optical field amplitude for mode m in the signal, *ψ*_*m*_ (*x,y*) is the mode profile of mode m, *v*_0_ is the optical frequency, and *θ*_*m*_ is the phase of mode m. Similarly, the total noise can be written as

2 $$e(x,y,t)=\mathop{\sum }\limits_{m=1}^{M}{C}_{{v}_{0},m}{{\rm{\psi }}}_{m}(x,y){e}^{j2\pi {v}_{0}t}$$

where *C*_*v*0,*m*_ is the optical field amplitude of the noise in mode m. Assuming that the electrical filter following square-law photodetection is an ideal integrate and dump circuit, the decision voltage is given by^6,9^

3 $$v(t)=\frac{1}{T}{\int }_{0}^{T}\iint dxdy{|S(x,y,t)+e(x,y,t)|}^{2}dt$$

where *T* is the bit period (or the symbol period for multilevel modulation formats). Since all the mode components are orthogonal to each other, the decision voltage in Eq. (3) can be written as

4 $$\begin{array}{rcl}v(t) & = & \mathop{\sum }\limits_{m=1}^{M}{E}_{{v}_{0},m}^{2}+\mathop{\sum }\limits_{m=1}^{M}{C}_{r{v}_{0},m}^{2}+\mathop{\sum }\limits_{m=1}^{M}{C}_{i{v}_{0},m}^{2}+2\mathop{\sum }\limits_{m=1}^{M}{E}_{{v}_{0},m}{C}_{r{v}_{0},m}\\  & = & \mathop{\sum }\limits_{m=1}^{M}{({E}_{{v}_{0},m}+{C}_{r{v}_{0},m})}^{2}+\mathop{\sum }\limits_{m=1}^{M}{C}_{i{v}_{0},m}^{2}\end{array}$$

where *C*_*rv*0_,_*m*_ and *C*_*iv*0_,_*m*_ are the in-phase (real) and quadrature (imaginary) noise components in mode m, which are zero-mean independent Gaussian random variables with a variance equal to noise power in mode m. Assuming that the photon number in each mode is the same, the decision voltage obeys the noncentral chi-squared distribution^10^, with variance *σ*^2^ = *n*_*sp*_*hυ*_0_*G*/*T*, noncentrality parameter ${m}_{c}^{2}=\mathop{\sum }\limits_{m=1}^{M}{{\rm{E}}}_{{v}_{0},m}^{2}=\frac{4{n}_{p}h{\upsilon }_{0}G}{T}$, and degree of freedom *n* = 2 *pM* (*p* = 1 when polarization filtering is used, otherwise *p* = 2), where *n*_*sp*_ is the population inversion factor (*n*_*sp*_ ≥ 1) of the optical amplifier, *n*_*p*_ is the average received photon number per bit period, *h* is the Planck constant, and *G* is the gain of the amplifier, which is assumed to be the same for all modes.

The probability density function (pdf) of noncentral chi-squared distribution is given by^10^

5 $$P(x/n,{\sigma }^{2},{m}_{c}^{2})=\frac{1}{2{\sigma }^{2}}{(\frac{x}{{m}_{c}^{2}})}^{n/4-0.5}\exp (-\frac{x+{m}_{c}^{2}}{2{\sigma }^{2}}){I}_{n/2-1}(\frac{\sqrt{{m}_{c}^{2}x}}{{\sigma }^{2}})$$

where *I*_*n*/2–1_ denotes the modified Bessel function of order *n*/2–1. After normalization, the variance is *σ*^2^ = *n*_*sp*_ and the noncentrality parameter is ${m}_{c}^{2}=4{n}_{p}$. Thus, the decision voltage of the ‘1’ and ‘0’ bits with polarization filtering can be written as *P*_1_(*x*/2*M*,*n*_*sp*_,4*n*_*p*_) and *P*_0_(*x*/2*M*,*n*_*sp*_,0). The bit error ratio (BER) of intensity modulation with direct-detection (IMDD) is given by^6^

6 $$BER=\frac{1}{2}({\int }_{0}^{{x}_{thre}}{P}_{1}(x){\rm{dx}}+{\int }_{{x}_{thre}}^{\infty }{P}_{0}(x){\rm{dx}})$$

where the decision threshold *x*_thre_ is determined by equating *P*_1_(*x*_thre_) = *P*_0_(*x*_thre_). The BER as a function of the received photon number per bit can be plotted numerically, thus the sensitivity for achieving a certain BER can be obtained. Figure 1(c) shows the sensitivity at a BER of 1 × 10^−9^ as a function of the number of modes.

For SM pre-amplified receivers, signal-ASE beat noise dominates over ASE-ASE beat noise. As the number of modes supported by the FM amplifier increases to accommodate moderate and stronger turbulence, the contribution of ASE-ASE beat noise increases even though the signal-ASE beat noise for a fixed total signal power is independent of the number of modes due to orthogonality of spatial modes. Nevertheless, as can be seen from Fig. 1(c), the sensitivity increases sub-linearly with the number of modes. The reason is that as the required number of photons/bit increases with the number of modes in the receiver, signal-ASE beat noise continues to dominate over ASE-ASE beast noise in the FM pre-amplified receivers. As a result, the sensitivity of a 50-mode (moderate turbulence) pre-amplified receiver has a sensitivity of 75 photons/bit, which represents a < 3 dB penalty compared to an ideal SM pre-amplified receiver.

In the analysis above, we also assume that the gain for each mode is the same. Optimally, a FM pre-amplified receiver should adjust the gain of each mode to be proportional to the power contained in that mode, similar to the principle of maximum-ratio combining^11^. However, this would entail a complicated amplifier design and control mechanism, which is counter to the desire for simplicity and reliability. In the experiments to follow, we take the approach of ensuring equalized modal gain to balance sensitivity, simplicity and reliability.

## FSO Experiment

**Figure 2:**
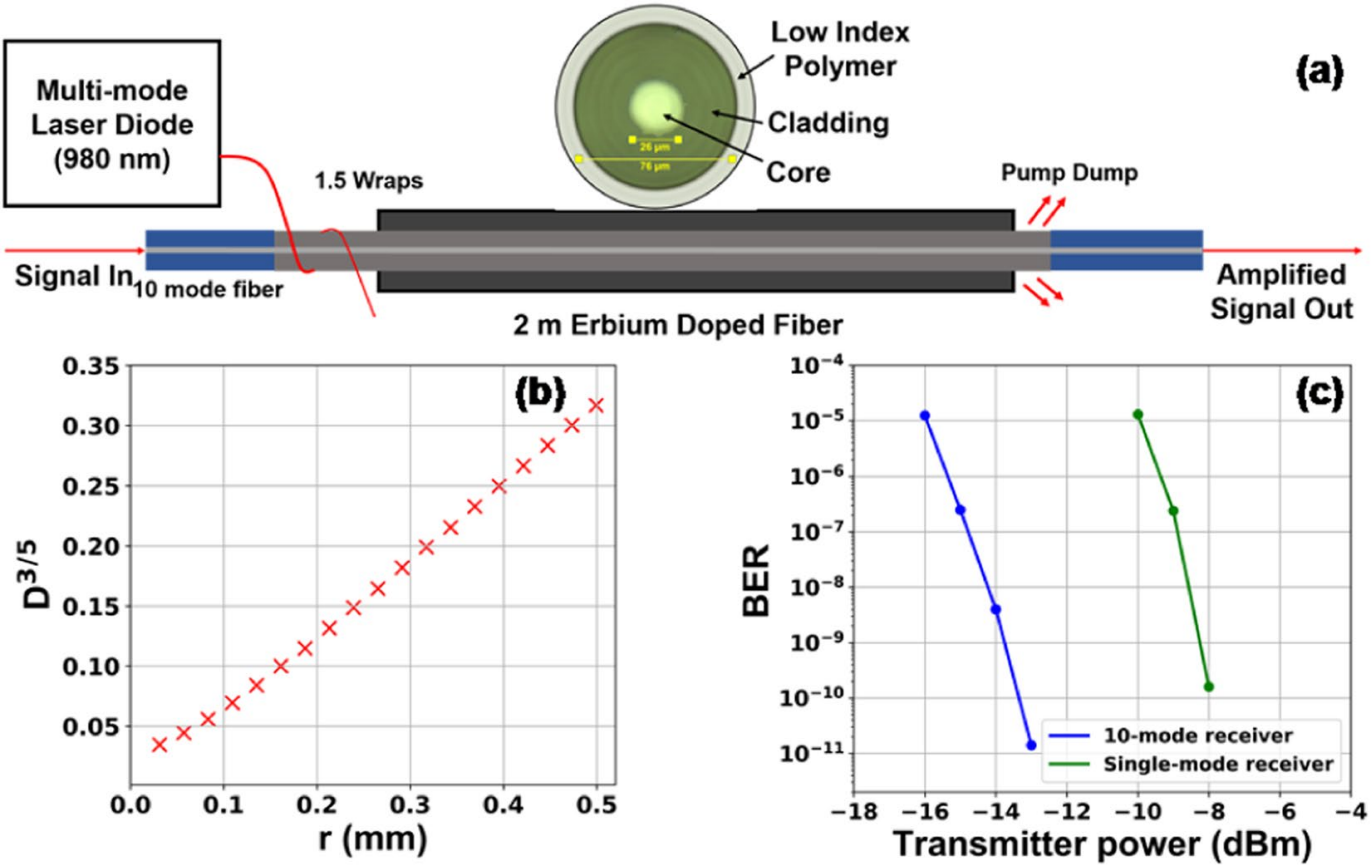
(**a**) Schematic of the FM preamplifier, (**b**) phase function of the phase plate emulating turbulence and (**c**) measured BERs as functions of transmitter power for SM and FM pre-amplified receivers.

We now describe our experimental results of FSO communication using the proposed FM pre-amplified receiver, in comparison with a SM pre-amplified receiver without AO, through an FSO channel with turbulence satisfying the Kolmogorov distribution. The schematic of the 10-mode cladding-pumped EDFA used in the experiment, shown in Fig. 2(a), has an Er-doped fiber (EDF) of core diameter 26 µm which can support 42 spatial modes for equalizing the gain of the 10 lowest-order modes^12^. At a pump power of 6.6 W, the average small-signal gain of the amplifier is 15 dB and the mode dependent gain (MDG) is less than 2 dB.

To emulate turbulence with a Kolmogorov distribution, we fabricated phase plates by repeatedly spray-coating glass substrates with acrylic^13,14^. We measured the phase structure function $D(r)=\langle {(\phi (\overrightarrow{r^{\prime} })-\phi (\overrightarrow{r^{\prime} }+\overrightarrow{r}))}^{2}\rangle$, where *φ* denotes the local phase, using phase-shifting interferometry, and *r* is the distance between two phase positions. The Kolmogorov model has the specific form of the phase structure function $D(r)=6.88{(\frac{r}{{r}_{0}})}^{5/3}$, where *r*_0_ is the Fired parameter representing the coherence length. We subsequently calculate *D*(*r*)^3/5^ as a function of *r*. The linear relationship shown in Fig. 2(b) validates the Kolmogorov distribution of the phase plates. For the phase plate that we used for our FSO experiment, *r*_0_ is calculated to be 5 mm.

A 10 Gb/s OOK signal beam from a SM transmitter is expanded into a diameter *d* around 1 cm and propagated through the phase plate, resulting in wavefront distortion of approximately ±4π across the beam. The BERs were measured at different transmitter powers as shown in Fig. 2(c). The results indicate that the 10-mode pre-amplified receiver provided a 6 dB gain in power budget over the SM pre-amplified receiver.

**Figure 3:**
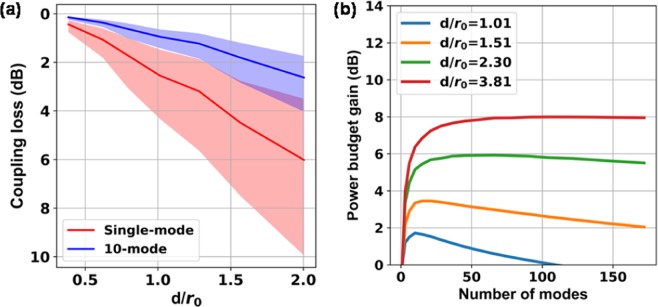
(**a**) Coupling loss of a distorted wavefront into a SM and FM fiber, as a function of *d*/*r*_0_ and (**b**) Power budget gain of FM over SM pre-amplified receiver as a function of the number of modes.

We now present the statistical property of the receivers based on simulations. In Fig. 3(a) we plot the coupling losses of the SM and FM receivers as functions of *d*/*r*_0_ for a fixed beam diameter of 1 cm, where different *d*/*r*_0_ values represent different turbulence levels. At each *d*/*r*_0_ value, we generate 200 wavefront distortions that follow the Kolmogorov model. The shaded region represents the standard deviation of power fluctuation for different realizations of each turbulence condition. It is observed that the average loss and received power fluctuation for the 10-mode receiver are much smaller than those for the SM receiver. In Fig. 3(b), we combined the results in Figs 1(c) and 3(a) to plot the gain in power budget for the FM pre-amplified receiver over that of the SM pre-amplified receiver as a function of the number of modes. The power budget for each case is set to ensure that the probability that the received power is below the sensitivity of the receiver is less than a desired outage probability. Based on the limited number of statistically realizations used in our simulation, we set the outage probability to 15.9%, that is, the received power is at most one standard deviation below the mean. As the number of modes increases, the (standard deviation of) received power (decreases) increases, which improves system performance. In the meantime, the receiver noise (sensitivity) increases (deteriorates). These two opposing dependences result in the existence of an optimum number of modes for each turbulence level.

## Comparison With Adaptive Optics

As shown in last section, FM pre-amplified receivers can increase the link power budget despite a penalty in received sensitivity as compared to that of SM pre-amplified receivers. This is because FM pre-amplified receivers can reduce the coupling loss to a larger degree than the penalty in sensitivity. As mentioned in the Introduction, adaptive optics is widely used to compensate wavefront distortions. It would be informative to compare the coupling efficiencies of distorted wavefronts into a FM pre-amplified receiver without AO and a SM pre-amplified receiver with AO.

**Figure 4:**
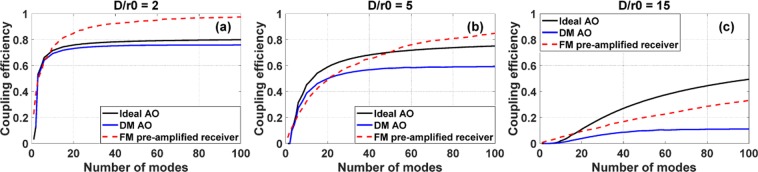
Coupling efficiencies using ideal AO, DM AO, or the FM pre-amplified receiver, with different levels of turbulence for (**a**) *d*/*r*_0_ = 2; (**b**) *d*/*r*_0_ = 5; (**c**) *d*/*r*_0_ = 15. The intensity is assumed to be uniform across the beam.

For AO based on deformable mirrors (DMs), Zernike modes are widely used to decompose the distorted wavefront when the aperture is circularly symmetric^15^. This is because the convergence speed is faster when the Zernike coefficients instead of the entire pixelated data from the wavefront sensor are used to control all DM actuators^16^. So simulating coupling efficiency as a function of the number of corrected Zernike modes is very relevant. The results of coupling efficiency as a function of the number of corrected Zernike modes for SM fiber with AO or the number of fiber modes for FM fiber without AO are shown in Fig. 4, corresponding to weak, moderate and strong turbulence, respectively. For AO, we include 1) ideal AO, in which wavefront distortion up to a certain order of Zernike modes are completely corrected, and 2) DM AO using a 12 × 12 deformable mirror. The input wavefront is generated using the power spectral density for turbulence in Kolmogorov’s model^13^:

7 $$\Phi (\kappa )=0.023{r}_{0}^{-5/3}{\kappa }^{-11/3}$$

where *k* is the spatial frequency. All results in Fig. 4 are averaged over 20 realizations with the same *d*/*r*_0_ values.

Under weak atmospheric turbulence (*d*/*r*_0_ = 2) in Fig. 4(a), all cases have similar coupling efficiencies for a small number of corrected Zernike modes or fiber modes (N < 10). For a larger number of modes, the coupling efficiencies for ideal AO and DM AO have a rather small difference, and FM pre-amplified receiver outperforms AO. Both AO approaches yield similar results because the distorted wavefronts under weak turbulence largely consist of lower-order Zernike modes. Under such conditions, the fitting errors using DM are small. The FM pre-amplified receiver can outperform AO because a superposition of a large number of fiber modes can match the uniform intensity across the beam, in addition to the distorted phase, while AO can only match the phase distortion. Under moderate atmospheric turbulence (*d*/*r*_0_ = 5) in Fig. 4(b), the differences in coupling efficiency between the two AO approaches become larger, since fitting errors for higher-order Zernike modes become larger using DM AO^17^. The FM pre-amplified receiver can outperform ideal AO for a large number of modes (N > 40). This is because the mode spectrum in the fiber-mode basis is more spread out compared with Zernike modes. Under strong atmospheric turbulence (*d*/*r*_0_ = 15) in Fig. 4(c), the differences in coupling efficiency between ideal AO and DM AO become even larger, and the FM pre-amplified receiver cannot outperform ideal AO. However, for all turbulence levels, the FM pre-amplified receiver can always outperform AO using a 12 × 12 deformable mirror.

The above simulations are based on the assumption that there is only phase distortion, which is only valid for weak atmospheric turbulence^1^. We now include the effect of intensity fluctuation due to strong atmospheric turbulence. In particular, we investigate the effects of turbulence on an FSO system with a range of 1 km and a ${C}_{n}^{2}$ value of 10^−12^, which exists frequently near ground in the middle of the day. The Fried parameter *r*_0_ is calculated to be 0.89 cm^18^ and the corresponding *d*/*r*_0_ value is 5.7 for a 2-inch receiving aperture. The intensity correlation length *ρ*_0_ can be calculated using *ρ*_0_ = *r*_0_/2.1, which is valid for the high ${C}_{n}^{2}$ value and scintillation index^19^.

**Figure 5:**
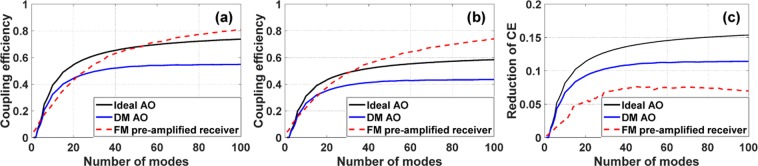
Coupling efficiencies using ideal AO, DM AO, or the FM pre-amplified receiver including (**a**) the effects of phase distortion only, (**b**) the effects of both phase and amplitude distortions for an FSO system with a propagation distance of 1 km and ${C}_{n}^{2}$ value of 10^−12^. (**c**) The corresponding reductions in coupling efficiency due to intensity fluctuations across the beam.

Under the above turbulence condition, the coupling efficiencies as functions of the number of corrected Zernike modes or fiber modes are shown in Fig. 5(b). The coupling efficiencies with the same phase distortions but ignoring intensity fluctuations are plotted in Fig. 5(a). The reductions in coupling efficiency due to the presence of intensity fluctuation for three cases are also shown in Fig. 5(c). The reductions in coupling efficiencies using AO are larger, since AO can only compensate distorted phase while the FM pre-amplified receiver is tolerant to both phase and amplitude distortions.

In addition, perfect wavefront sensing has been assumed in above simulations for AO. However, in practice, the accuracy of Shack-Hartmann wavefront sensors degrades severely for moderate and strong turbulence due to scintillations^20^. Interferometric wavefront sensors are required in this case^18,21^, but are not yet commercially available. Turbulence can also cause beam wandering and associated fluctuations in received signal. An additional pointing and tracking system will be needed for both the FM pre-amplified receiver and the SM receiver with AO^22^.

## Conclusions

In conclusion, we propose and demonstrate FM pre-amplified receivers for FSO to achieve high sensitivity, simplicity and reliability by taking advantage of recent advances in SDM. In this paper, only results for OOK are presented, but the technique can be easily extended to other direct-detection modulation formats, such as differential phase-shift keying (DPSK). Even though multi-subaperture based digital coherent detection can potentially combat turbulence^23^, the implementation is complex and costly. Comparison with adaptive optics using deformable mirrors also shows the coupling-efficiency advantage of the proposed method. The above reasons suggest that the technique presented here likely represents an advantageous, practical method of combating turbulence in FSO.

## Methods

In the FSO experiment, the FM EDF has an outer cladding with lower refractive index and an inner cladding with higher refractive index. Pump light coming from a multi-mode laser diode (MMLD) is coupled into the inner cladding of the EDF using side pumping. To do so, we spliced the multi-mode fiber (MMF) pigtail of the MMLD to a coreless fiber and down tapered the coreless fiber from 125 μm to 20 μm in a tapering length of 30 mm. The high-power MMLD is from BWT, and the signal is detected by a multimode (MM) InGaAs PIN + TIA receiver spliced to a MM pigtail fiber.

For the simulation in Fig. 3, a graded-index (GRIN) few-mode fiber (FMF) with core radius of 14 μm and NA of 0.17, is used. For the simulations in Figs 4 and 5, a GRIN MMF with core radius of 25 μm, and NA of 0.21, is used. The magnification of the imaging system is adjusted for different turbulence levels in the simulation of comparison with AO. As shown in Fig. 1, a lens is used to focus the free-space beam onto the facet of the fiber, and the focal length of the lens or the magnification of the imaging system affects the coupling efficiency^24^. For coupling a uniform field into the fiber, an optimum magnification can be calculated^4^, which was adopted when simulating the performance of AO. When the turbulence level increases, the number of spatial modes contained in the distorted wavefront and in the receiving FMF also increase. In the meantime, the effective areas of free-space modes and fiber modes scale differently. As a result, an optimum magnification exists for coupling a certain number of free-space modes into the receiving FMF. For the results presented below, an optimum magnification corresponding to the number of free-space modes was used for simulating the performance of the FM pre-amplified receiver. This is reasonable because FSO systems will be designed for the worst-case scenario, i.e., the largest number of modes.

## Data Availability

All data generated or analysed during this study are included in this published article.
